# Potential natural inhibitors of xanthine oxidase and HMG-CoA reductase in cholesterol regulation: *in silico* analysis

**DOI:** 10.1186/s12906-020-03162-5

**Published:** 2021-01-01

**Authors:** Rishab Marahatha, Saroj Basnet, Bibek Raj Bhattarai, Prakriti Budhathoki, Babita Aryal, Bikash Adhikari, Ganesh Lamichhane, Darbin Kumar Poudel, Niranjan Parajuli

**Affiliations:** 1 Central Department of Chemistry, Tribhuvan University, Kirtipur, Kathmandu Nepal; 2 Center for Drug Design and Molecular Simulation Division, Cancer Care Nepal and Research Center, Jorpati, Kathmandu Nepal

**Keywords:** Natural products, Molecular docking, Cholesterol, Enzyme inhibition

## Abstract

**Background:**

Hypercholesterolemia has posed a serious threat of heart diseases and stroke worldwide. Xanthine oxidase (XO), the rate-limiting enzyme in uric acid biosynthesis, is regarded as the root of reactive oxygen species (ROS) that generate atherosclerosis and cholesterol crystals. β-Hydroxy β-methylglutaryl-coenzyme A reductase (HMGR) is a rate-limiting enzyme in cholesterol biosynthesis. Although some commercially available enzyme inhibiting drugs have effectively reduced cholesterol levels, most of them have failed to meet potential drug candidates’ requirements. Here, we have carried out an *in-silico* analysis of secondary metabolites that have already shown good inhibitory activity against XO and HMGR in a wet lab setup.

**Methods:**

Out of 118 secondary metabolites reviewed, sixteen molecules inhibiting XO and HMGR were selected based on the IC_50_ values reported in in vitro assays. Further, receptor-based virtual screening was carried out against secondary metabolites using *GOLD Protein-Ligand Docking Software*, combined with subsequent post-docking, to study the binding affinities of ligands to the enzymes*. In-silico* ADMET analysis was carried out to explore their pharmacokinetic properties, followed by toxicity prediction through ProTox-II.

**Results:**

The molecular docking of amentoflavone (GOLD score 70.54, ∆G _calc._ = − 10.4 Kcal/mol) and ganomycin I (GOLD score 59.61, ∆G _calc._ = − 6.8 Kcal/mol) displayed that the drug has effectively bound at the competitive site of XO and HMGR, respectively. Besides, 6-paradol and selgin could be potential drug candidates inhibiting XO. Likewise, n-octadecanyl-O-α-D-glucopyranosyl (6′ → 1″)-O-α-D-glucopyranoside could be potential drug candidates to maintain serum cholesterol. *In-silico* ADMET analysis has shown that these sixteen metabolites were optimal within the categorical range compared to commercially available XO and HMGR inhibitors, respectively. Toxicity analysis through ProTox-II revealed that 6-gingerol, ganoleucoin K, and ganoleucoin Z are toxic for human use.

**Conclusion:**

This computational analysis supports earlier experimental evidence towards the inhibition of XO and HMGR by natural products. Further study is necessary to explore the clinical efficacy of these secondary molecules, which might be alternatives for the treatment of hypercholesterolemia.

## Background

Globally, ischemic heart disease (IHD) is the foremost cause of cardiovascular diseases (CVDs) followed by stroke [1]; also, high cholesterol level is accountable for one-third of all cases of IHD and increased risks of stroke too [2]. Cholesterol acquired via de novo synthesis (600–900 mg/day) & diet (300–500 mg/day), transported via blood, and excreted through bile acid biosynthesis (500–600 mg/day) and as biliary cholesterol (600 mg/day) are the major aspects of its homeostasis in human [3, 4]. Several elements, such as age, gender, human genetics, dietary habits, physical activity, and metabolic disorder, have affected cholesterol levels [5]. Nonetheless, various epidemiological studies have recently confirmed that plasma cholesterol levels are correlated with many bacterial gut microbiomes [6]. Cholesterol is an indispensable structural component of the cell membrane and serves as the substrate for biosyntheses of vitamin D, bile acids, and steroid hormones [7]. However, its accumulation in the body is associated with atherosclerosis, hypertension, and ultimately to CVDs, which have resulted in increased mortality and morbidity rates globally [1, 8]. Whence, from a therapeutic point of view, the regulations of total serum cholesterol and triglycerides have gained much heed against hyperlipidemia [9].

Statins, competitive inhibitors of HMGR, are the widely recognized medication to lower cholesterol levels [10, 11]. Other available medications include ezetimibe (cholesterol absorption and Niemann-Pick C1-like protein inhibitor) and bile acid sequestrants (induce hepatic conversion of cholesterol into bile acids) [12, 13]. Lomitapide (microsomal triglyceride transfer protein inhibitor), mipomersen (apolipoprotein B 100 inhibitor), alirocumab & evolocumab (proprotein convertase subtilisin kexin type 9 (PCSK9) inhibitors), and bempedoic acid (adenosine triphosphate-citrate lyase inhibitor) are the latest approved remedy against hypercholesterolemia in the last decades [14–16]. Moreover, the inhibitors of acyl-CoA cholesterol acyltransferase 2 (ACAT-2), diglyceride acyltransferase 2 (DGAT-2), high-density lipoproteins (HDL) modulating drugs, small interfering RNA (inclisiran), and angiopoietin-like protein 3 (ANGPTL3) are under progress for clinical trials in human. They could be prospective in lowering cholesterol levels [11, 17].

XO (290 kDa) is involved in uric acid biosynthesis and is regarded as the root of ROS (H_2_O_2_ and O_2_^−^) in vascular tissue and, hence engenders atherosclerosis [18–20]. It is the rate-limiting enzyme involved in the catabolism of purine nucleotides, the step that oxidizes xanthine to uric acid [21, 22]. IHD correlates with upraised uric acid levels, and XO inhibitors such as allopurinol and febuxostat have palliated the risk of IHD by minimizing the effect of ROS and enhancing endothelial function and ATP synthesis in ischaemic tissue [23, 24]. Elevated cholesterol level increases the activity of XO that causes oxidative stress in tissue and decreases the activity of nitric oxide synthase (NOS) that surges CVDs risks [25, 26]. This oxidative stress converts low-density lipoprotein (LDL) to oxidized LDL, which is absorbed by macrophages in the intima of the vascular wall that eventually forms cholesterol crystals and deteriorates endothelial function [27, 28]. In cholesterol biosynthesis, the conversion of acetyl CoA to HMG-CoA is catalyzed by HMGR (200 kDa) found in the endoplasmic reticulum (ER) [29, 30]. It is the rate-limiting enzyme involved in the synthesis of mevalonate, while the post-squalene portions are regulated by cytochrome P450 51 [3].

The mechanisms, pharmacokinetics, interactions, and side effects of the drugs, as mentioned above, are well explained by Fein gold [31]. On account of side effects, it is challenging to explore new drugs of high medicinal importance. In this study, we have focused on potential HMGR and XO inhibitors, based on natural products, which are considered as the wellspring of biologically and pharmacologically active sources of secondary metabolites [32, 33]. We have performed virtual screenings of some secondary metabolites showing good inhibitory activity with the aid of computer-based computation. *In-silico* ADMET analysis, which would lower the use of animal testing following ethical guidelines in the pharmacological experiment, were studied using the pKCSM web application. Furthermore, toxicity analysis through ProTox-II and molecular docking using *GOLD Protein-Ligand Docking Software* combined with subsequent post-docking were carried out to uncover further evidence on the inhibition mechanism. We believe that our findings would be beneficial in drug development programs concerning hypolipidemic agents.

## Methods

### Selection of XO and HMGR

The crystal structures of XO (PDB ID:1N5X, 2.80 Å) and HMGR (PDB ID:1HWK, 2.22 Å) were obtained from Protein Data Bank (PDB) [34] and were chosen for their availability as refined crystal structures, which were confirmed by X-ray diffraction [35] [29]. The crystal structure of XO, complexed with febuxostat, was retrieved to understand the protein-ligand docking algorithm and to predict the position of metabolites in the binding cavity of XO. The structure of XO was a homodimer (chain A and B), where only chain A was used for docking studies. Similarly, the dimeric crystal structure of HMGR, complexed with atorvastatin, was used for docking studies where two neighboring monomers were relevant for making interactions with statins [35, 36]. Other chains and water molecules were removed using MOE protein preparation wizard [37].

### Designing of ligands

The data set was prepared based on an extensive literature survey taking IC_50_ values of in-vitro enzyme inhibition assays against XO and HMGR by various secondary metabolites. Based on IC_50_ values, sixteen plant- and fungus-based secondary metabolites (Tables 1 and 2**)** were chosen for the ligand-protein docking study. The docking study was performed against commercial drugs such as atorvastatin, simvastatin, lovastatin, and pravastatin for HMGR. On the other hand, commercial drugs such as allopurinol, febuxostat, topiroxostat, and probenecid were used for molecular docking studies with XO. The structures of the ligand molecules and the control drugs of both enzymes were retrieved from the PubChem database [38] and verified from SciFinder. The structures were retrieved in SDF format and were converted to PDB and MOL2 format using Discovery Studio Visualizer 4.0 software. The structure and complete chemical properties, torsional energy, van der Waals potential energy, electrostatic energy, weight, log *P*, total polar surface area (TPSA), donor atoms, and acceptor atoms of the ligands were listed (Supplementary Table 4S) by the help of MOE Module [39].

### Prediction of active sites

Amino acids involved in active pocket formation were determined using Site-Finder (Supplementary Table 3S), which calculates possible active sites in a receptor from the 3D atomic coordinates based on alpha shape methodology [40]. All the amino acid residues were listed adequately from the active site analysis and validated from published crystal structure active residues and published research journals for complete study [41, 42].

### Computational analysis

Flexible docking simulations were performed using GOLD [43] to investigate the molecules’ binding modes to predict the efficiency of secondary metabolites to inhibit HMGR and XO enzymes. These novel potential compounds were obtained from the extensive literature review and deposited in the inbuilt CHEM-TU Natural Metabolites Library. Genetic algorithms had been used in GOLD that had integrated fully- and partially- ligand flexibility docking approaches in the neighborhood of the protein’s active site [44] to determine the appropriate binding positions, orientations, and conformations of ligands [45]. All other parameters were maintained as default. In the flexible docking process and according to the GOLD score molecular mechanics function, the ligand with the highest fitness GOLD score was deemed to have the highest binding affinity. The function was expressed as,

$$ \mathrm{GOLD}\ \mathrm{Fitness}=\mathrm{Shb}\_\operatorname{ext}+1.375\ \left(\mathrm{Svdw}\_\operatorname{ext}\right)+\mathrm{Shb}\_\operatorname{int}+\mathrm{Svdw}\_\operatorname{int}\Big) $$

Where Shb_ext is the protein-ligand hydrogen-bond score, and Svdw_ext is the protein-ligand van der Waals score. Shb_int contributes to fitness due to intramolecular hydrogen bonds in the ligand, Svdw_int is the contribution due to intermolecular strain in the ligand. The details about molecular docking results are mentioned in Supplementary Table 1S.

### Estimation of binding energy

We applied a semi-empirical method in-built to AutoDock Vina [46]. The prediction of absolute binding energies may be less accurate than more computationally expensive, purely force field-based methods, but this semiempirical approach is considered well-suited for the relative rankings [47]. The pIC_50_ value was calculated using formula: pIC50 = −log (IC50*10^− 9^) and ∆G _Experimental_ was calculated by the equation: **∆G**_**Exp.**_ = − RT ln (pIC_50_) [48].

### Prediction of ADMET profiles

Drug discovery programs assisted analysis of absorption, distribution, metabolism, excretion, and toxicity (ADMET) properties of secondary metabolites. The potential pharmacokinetic properties prediction was completed using the pKCSM web application [49]. *In-silico* potential toxicity of secondary metabolites was assessed by ProTox-II, which was based on toxic, lethal dose (LD_50_) value ranging from class 1 and 2 (fatal), class 3 (toxic), class 4 and 5 (harmful), while class-6 (non-toxic) [50]. The confidence score of secondary metabolites for specific targets had been used to predict the reliability of toxicity based on the value of more than 0.7 [51].

## Results

**Fig. 1:**
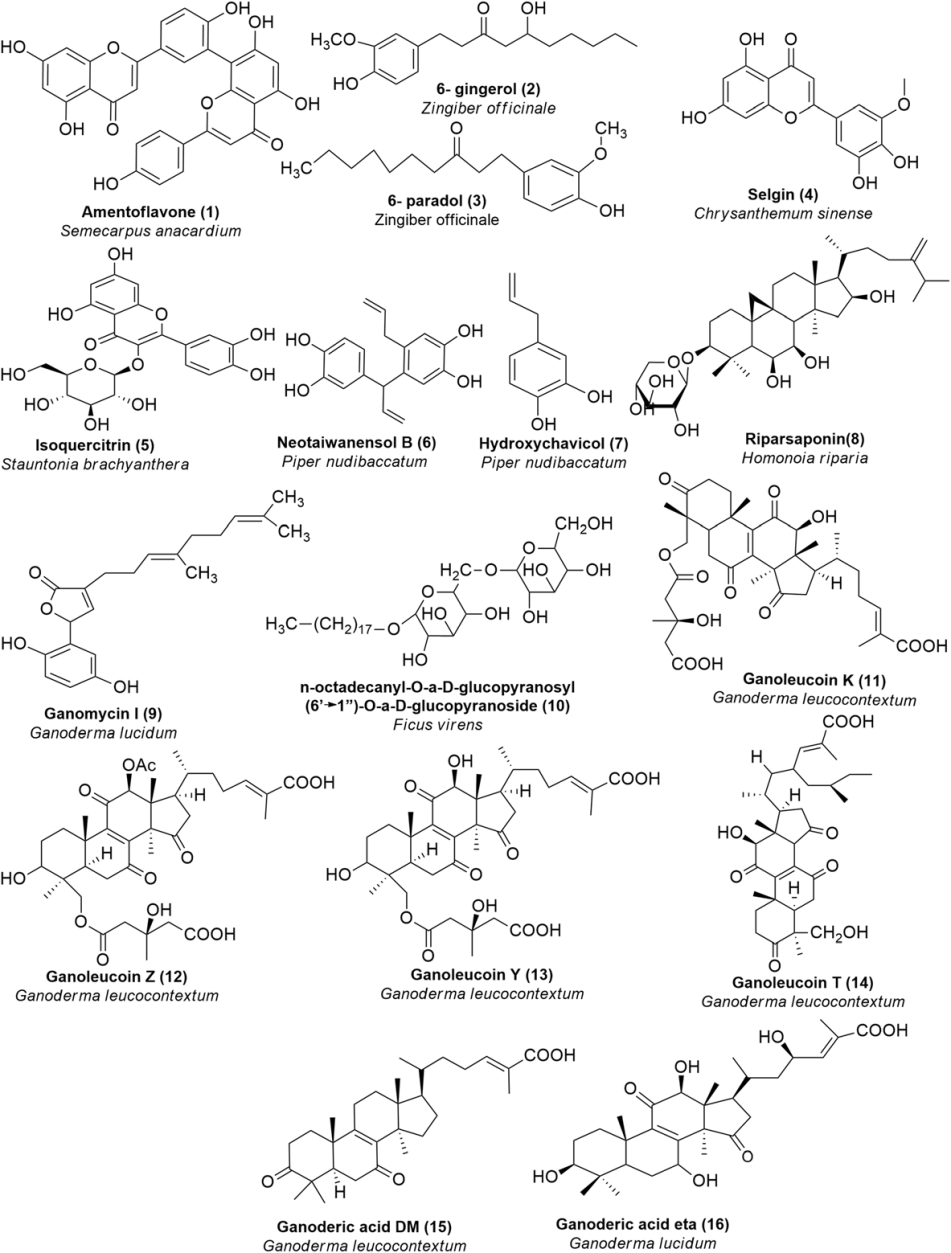
Plant and fungus-based secondary metabolites inhibiting XO (**1–8**) and HMGR (**9–16**)

**Fig. 2:**
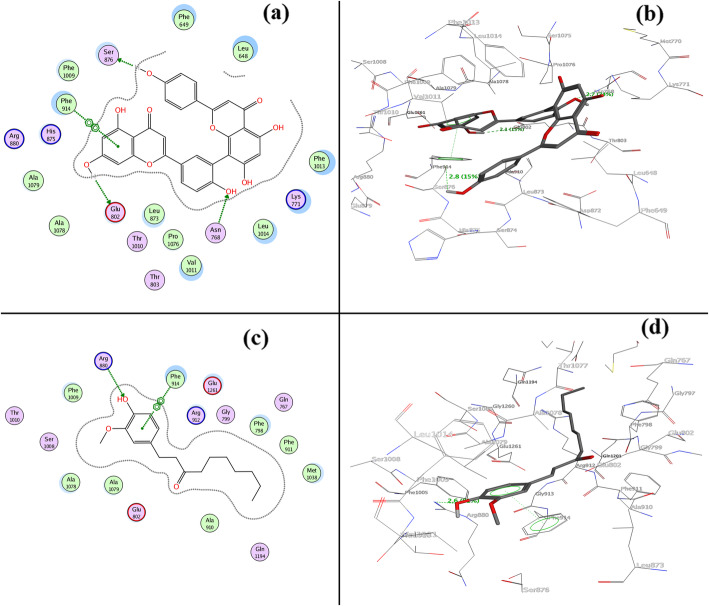
**a** 2D and **b** 3D interactions of XO with amentoflavone (1) (GOLD fitness score of 70.54); **c** 2D and **d** 3D (lower) interactions of XO with 6-gingerol (2) (GOLD fitness score of 68.75)

**Fig. 3:**
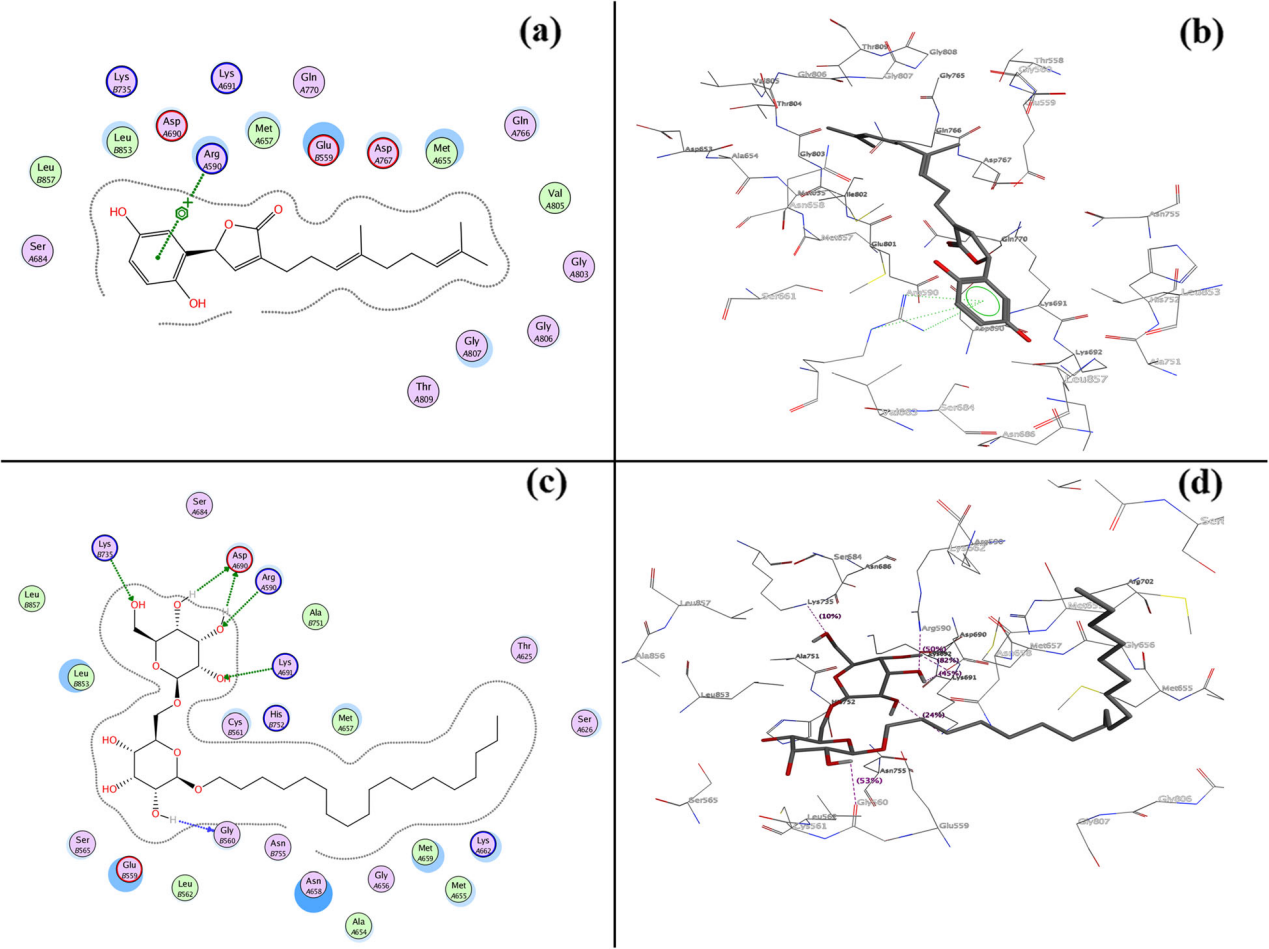
**a** 2D and **b** 3D interactions of HMGR with ganomycin-I (9) (GOLD fitness score of 59.61); **c** 2D and **d** 3D interactions of HMGR with n-octadecanyl-O-훂-D-glucopyranosyl(6′ → 1″)-O-훂-D-glucopyranoside (10) (GOLD fitness score of 52.69)

In the beginning, a dataset was prepared based on a literature review taking IC_50_ values of in-vitro enzyme inhibition assays with HMGR and XO by natural products. In this article, Supplementary Table 2S provides details about the targets and their description. Figure 1 presents the structure of secondary metabolites and natural sources, which were used in this study. Tables 1 and 2 give GOLD fitness scores and hydrogen bonding interaction values between targets and secondary metabolites, interaction type, and bond length of the docking. The 2D and 3D interactions of the high GOLD scoring metabolites and commercial drugs with the target enzymes were shown in Figs. 2 and 3 and Supplementary Figs. (1S, 2S, 3S, 4S, 5S, 6S, 7S). The molecular properties of commercial drugs and selected secondary metabolites were shown in Supplementary Table 4S. Supplementary Table 11S and 12S illustrates the theoretical (calculated) and experimental binding energy of secondary metabolites.

**Table 1:** GOLD Fitness score and Protein-Ligand Interactions of Protein ID: 1N5X with XO Inhibitors

Compounds	Reported IC_**50**_ value (μM)	GOLD Score	H-Bond Interaction Residues	Bond Length	Other Interacting Residues
Amentoflavone **(1)**	0.09	70.54	Glu 802 Asn 768 Phe 914 Ser 876	2.1 2.7 - 2.8	Pi-Pi interaction with Phe 914
6- Gingerol **(2)**	10.50	68.75	Thr 1010 Ala 1079 Phe 798 Phe 914	1.9 2.4 2.5 -	Pi-Pi interaction with Phe 914
6- Paradol **(3)**	12.40	67.34	Arg 880 Phe 914	3.2 -	Pi-Pi interaction with Phe 914
Selgin **(4)**	0.22	61.41	Phe 914 Glu 1261 Thr 1010 Arg 880	- 2.0 3.0 2.4/3.2	Pi-Pi interaction with Phe 914
Isoquercitrin **(5)**	1.60	55.4	Thr1010 Ala 1079 Phe 914 Ser 876 Asp 872 Asn 768 Lys 771	2.90/3.00 3.00 2.8 - 2.9 3 2.5	Pi-Pi interaction with Phe 914
Neotaiwanensol B **(6)**	0.28	52.77	Ser 876 Thr 1010 Arg 880 Phe 914	3 1.6 2.90/2.90 2.9	Pi-Pi interaction with Phe 914
Hydroxychavicol **(7)**	0.38	49.02	Arg 880 Phe 914	2.9/2.7 -	Pi-Pi interaction with Phe 914
Riparsaponin **(8)**	0.01	27.92	Leu 648	3.2	No any extra interaction

**Table 2:** GOLD Fitness score and Protein-Ligand Interactions of Protein ID: 1HWK with HMGR Inhibitors

Compounds	Reported IC_**50**_ value (μM)	GOLD Score	H-Bond Interaction Residues	Bond Length (Å)	Other Interacting Residues
Ganomycin I **(9)**	12.3 ± 1.7	59.61	Arg A590	–	Pi-cation interaction with Arg A590
n-octadecanyl-O-α-D-glucopyranosyl(6′ → 1″)-O-α-D-glucopyranoside **(10)**	0.164	52.69	Lys A691 Gly B560 Lys B735 Arg A590 Asp A690	3.1 2.8 3.2 2.9 1.7/3.3	No any extra interaction
Ganoleucoin K **(11)**	10.7 ± 2.9	43.91	Asn A658 Val A805	2.5 2.4	No any extra interaction
Ganoleucoin Z **(12)**	8.68 ± 0.96	43.41	Lys A691 Asp A690 Arg A590 Lys B735 Leu B857	2.8 2.10/ 3.30 2.7 3.1 2.2	No any extra interaction
Ganoleucoin Y **(13)**	9.72 ± 0.91	30.85	Asn A658 Ser A661	2.6 2.3	No any extra interaction
Ganoleucoin T **(14)**	10.3 ± 1.78	29.15	Asp A690 Gly B560 Thr B558	1.3 3.2 2.2	No any extra interaction
Ganoderic acid DM **(15)**	9.5 ± 1.5	18.89	Arg A590 Asn B755	2.7 3.0	No any extra interaction
Ganoderic acid η **(16)**	29.8 ± 1.5	12.13	Arg A590 Lys A692 Lys B735	2.5 2.9 2.8	No any extra interaction

### Molecular docking of ligands into XO

From the molecular docking, we observed electrostatic 2D and 3D molecular surfaces (Fig. 2**)**; the study showed that amentoflavone **(1)** and 6-paradol **(3)** were well located into the active site of XO with the GOLD fitness score of 70.54 and 67.34 **(**Table 1**)** respectively which is higher than commercial drugs febuxostat (GOLD score 64.53), topiroxostat (GOLD score 61.46), probenecid (GOLD score 57.75), and allopurinol (GOLD score 46.16) (Supplementary Table 10S).

Similarly, selgin **(4)**, isoquercitrin **(5)**, neotaiwanesol B **(6)**, and hydroxychavicol **(7)** have shown satisfactory interactions with the active site residues with fitness score of 61.41, 55.4, 52.77, and 49.02, respectively.

Amino acid residues participating in forming Pi-Pi and Pi-cation interactions were also investigated. Amentoflavone **(1)** and 6-paradol **(3)** was surrounded by several amino acid residues (Glu 802, Asn 768, Phe 914, ser 876, and Arg 880), which were described as active site residues [52]. It has been reported that Arg 880 and Glu 802 residues play a crucial role in the hydroxylation of substrate xanthine [53]. Hydrogen bonding with Glu 802 residue for a top-scoring amentoflavone **(1)** and Arg 880 for commercial drugs (febuxostat) was observed with the bond lengths of 2.1 Å and 3.0 Å, respectively. Nevertheless, 6-paradol **(3)**, selgin **(4)**, and isoquercitrin **(5)** also showed significant interactions through H-bonding within the range of 2–3.2 Å as well as Pi-Pi interaction with Phe 914 of target XO protein.

### Molecular docking of ligands into HMGR

Figure 3 showed the 2D and 3D molecular surface’s orientation of the top-scored secondary metabolites ganomycin I **(9)** and n-octadecanyl-O-α-D-glucopyranosyl (6′ → 1″)-O-α-D-glucopyranoside **(10)**. The study has shown that ganomycin I **(9)** bound competitively into the active site of HMGR with the GOLD fitness score of 59.61 **(**Table 2**),** higher than commercial drugs simvastatin (GOLD fitness score 56.81), lovastatin (GOLD fitness score 41.36), and pravastatin (GOLD fitness score 54.83) **(**Supplementary **Table 10S).** Similarly, n-octadecanyl-O-α-D-glucopyranosyl(6′ → 1″)-O-α-D-glucopyranoside **(10)** has shown satisfactory interactions with the active site residues with fitness scores of 52.69. Arg A590 amino acid was involved in forming Pi-cation interaction with ganomycin I (9) with unique features of high Vander Waals energy 32.11 (Supplementary **Table 4S**). In commercial drugs, other active residues (Arg A590, Ser A684, Gly A692, Lys A691, Asp A690, Glu B559, Lys B735) were involved in forming hydrogen bonds as wells as in Pi-Pi interactions. Furthermore, ganoleucoin T **(14)**, ganoderic acid DM **(15)**, and ganoderic acid η **(16)** were found interacting with target protein amino acid residues via H-bonds ranging 1.3–3.3 Å (Fig. 3).

### Analysis of binding energy

The relationship between different variables like binding free energies (theoretical and experimental), IC_50,_ and pIC_50_ of secondary metabolites for both XO and HMGR was studied. For XO, there is a positive (direct) relationship between theoretical and experimental binding energy (low degree: 0.198); experimental binding energy and IC_50_ (high degree: 0.869), and a negative (inverse) relationship between theoretical binding energy and pIC_50_ (low degree: − 0.186); experimental binding energy and pIC_50_ (high degree: − 0.998) and between IC_50_ and pIC_50_ (high degree: − 0.840). Similarly, for HMGR, there is a negative (inverse) relationship between theoretical and experimental binding energy and pIC_50_ (high degree: -0.999); IC_50_ and pIC_50_ (high degree: − 0.739) and a positive (direct) relationship between experimental binding energy; IC_50_ (high degree: 0.762) and between theoretical binding energy and pIC_50_ (moderate: 0.525).

### Analysis of ADMET profiles

Supplementary Table 8S **(a)** and **(b)** showed the detail of the ADMET analysis of sixteen metabolites. Compounds (**1)–(9)** and (**14)–(16)** were significantly absorbed, while (**10)–**(**13)** were found to be poorly absorbed in the human small intestine. However, these metabolites could not cross blood-brain barriers (BBB) readily, and none of this inhibited CYP3A4 and CYP2D6. Utterly, all the metabolites showed low hepatic and renal clearance. According to the *in-silico* toxicity prediction through Protox-II, compounds (**14**) and (**16**) were non-toxic while compounds (**3**), (**4**), (**5**), (**8**), (**1**), (**6**), (**7**), (**9**), (**10**), and (**15**) were classified under the harmful category. Compounds (**2**), (**11**), (**12**), and (**13**) were found to be toxic for human use. Moreover, Supplementary Table 7S showed the detail of predicted LD_50_ values and confidence scores of specific active targets of each metabolite. This analysis showed that all sixteen metabolites except (**2**), (**11**), (**12**), and (**13**) were optimal within the categorical range compared to commercially available XO and HMGR inhibitors, respectively.

## Discussion

Active plant-derived drugs can create a new era as therapeutic agents. Although this article is based on molecular docking of natural products already characterized, **Table 9S** shows crude extracts of natural sources that show significant inhibitory activity against XO and HMGR for broader coverage in the field. Molecular docking continues to hold great promise in the field of computer-based drug design. The higher the GOLD fitness score of ligands, the higher the binding capacity to protein residues [45]. Hydrogen bonding and hydrophobic interactions play an essential role in determining the binding affinity and stability of protein-ligand complexes [54, 55]. Furthermore, the lower the protein-ligand complex’s binding energy, the higher is its stability [56].

The GOLD fitness scoring of **(1)** and **(3)** was higher than commercially available XO inhibitors. Moreover, the GOLD fitness score of (**4**), **(5)**, **(6)**, and **(7)** are harmonious with that of commercial XO inhibitors. The binding energy of **(2)** and **(3)** was found lower than commercially available XO inhibitors illustrating its better stability. For instance, the inhibitory effect of the XO is probably due to the lodging of **(1)** in its active site via H-bonding with neighboring Glu 802, Asn 768, Ser 876, and Phe 914 amino acid residues and Pi-Pi interaction with Phe 914. From this interaction, the catalytic center of XO would undergo a conformational change and suppress the enzyme activity [57]. XO is a complex molybdoflavoprotein that produces ROS via reducing oxygen at the flavin center [58]. With the inhibition of XO, increased HDL levels have been reported in animal models [59]. Moreover, HDL is associated with reverse cholesterol transport and cholesterol efflux that prevents atherosclerosis and, ultimately, CVDs [60]. Moreover, the studies showed the superiority of non-purine analog of XO inhibitors over purine analogs regarding side effects and toxicity [61]. On account of this, the compounds **1–8** are non-purine analogs. Previously, **(1)** and **(3)** were shown with anticancer, anti-inflammatory, and antioxidant activities [62–65]. Thus, this is evident that XO inhibitors, used widely as the remedy of hyperuricemia, could be potential candidates for regulating body cholesterol levels.

Similarly, the binding affinity of **(9)** to enzyme active sites, via H-bonding and Pi-Pi interaction with Arg A590 amino acid residue, is higher than that of commercial inhibitors of HMGR. Moreover, compound **(9)**, **(10)**, and **(11)** showed lower binding energy than commercially available HMGR inhibitors, which further strengthens the stability of the protein-ligand complex thus formed. The probable mechanism is competitive inhibition via the binding of **(9)** and **(10)** at the active site of HMGR inhibiting natural substrate (HMG-CoA). This inhibition lowers the cholesterol levels in the ER and induces the transport of sterol regulatory element-binding proteins (SREBPs) to the Golgi body. These SREBPs express the genes of LDL receptors and speed up removing LDL and VLDL from plasma [31, 66]. Previously, **(9)** had been shown as HMGR and an α-glucosidase inhibitor [67]. This showed that these secondary metabolites also bind effectively to enzymes like commercial drugs and could be potential inhibitors of XO and HMGR, respectively. Though the compounds **(2)** and **(11)–(13)** possess appropriate GOLD scores, they are not regarded as potential drugs owing to their toxicity on virtual screening.

In ADMET profiles, an intestinal absorption value above 30% signifies good absorption in the human intestine. The volume of distribution (VDss) is taken into consideration if the logVDss value greater than 0.45. The compounds with logBB < − 1 are said to be poorly distributed to brain, while those having logBB > 0.3 are potential to cross BBB [49, 68, 69]. The cytochrome P450 (CYP) plays a major role in drug metabolism with CYP (1A2, 2C9, 2C19, 2D6, and 3A4), mainly responsible for the biotransformation of greater than 90% of drugs in phase-1 metabolism [70, 71]. However, among these P450 families, CYP3A4 is the focus part of this study [72]. The relationship between the rate of elimination of the drug and the drug concentration in the body is best described by total clearance [73]. Moreover, it is mandatory to examine the toxicity value based on parameters like ames toxicity, hepatotoxicity, and oral toxicity range because these play a critical role in the selection of drugs.

As discussed earlier, the studies suggest that XO inhibitors lessen the threat of major adverse cardiovascular events (MACE) [74]. With the availability of a handful of XO inhibitors in clinical use for treating hyperuricemia, the novel drugs addressing cardiac complications are demanding [75]. Recently, non-purine-like XO inhibitors have drawn significant attention, so they do not interfere with other facets of purine metabolism [52]. XO inhibitors could be equally useful for diabetic patients [75]. Although statins have been used widely for many decades as HMGR inhibitors, they are marked with muscular disorders, diabetes, liver diseases, etc. [31, 76]. Novel HMGR inhibitors are called for minimizing these side effects. However, the problems associated with natural secondary metabolites, such as their stability, solubility, and bioavailability, need to be considered to use them as therapeutic agents [77]. Furthermore, clinical trials, structural modifications, and biomimetic synthesis may lead to the discovery of promising inhibitors of HMGR and XO, which could contribute to the treatment of hyperuricemia and lowering cholesterol.

## Conclusions

The most potent natural inhibitors of the XO and HMGR are selected based on reported experimental IC_50_ values and show that these metabolites could be potent in lowering cholesterol levels. The molecular docking analysis shows that amentoflavone **(1)**, 6-paradol **(3)**, and selgin **(4)** fit well in the binding site of XO and lower the catalytic activity of the enzyme by changing its conformation. The studies and evidence suggest that XO inhibitors could be potential in regulating cholesterol levels. Similarly, the binding affinity of ganomycin-I **(9)** and n-octadecanyl-O-α-D-glucopyranosyl(6′ → 1″)-O-α-D-glucopyranoside **(10)** are involved in arresting the cholesterol biosynthetic pathway. Thus, the shreds of evidence like experimental IC_50_ value, computational docking, *in-silico* pharmacokinetics, and toxicity analysis proclaim that natural products could be used to develop potential future drug candidates.

## Supplementary Information

**Additional file 1: Table** **1****S.** Details about molecular docking platform. **Table** **2****S.** List of Targets showing the PDB ID, resolution and description of the proteins selected for docking with complexed inhibitor. **Table 3S**. Active Site residues of HMG-CoA Reductase and Xanthine Oxidase. **Table 4S.** Molecular Properties of standard compounds and selected secondary metabolites. **Table 5S.** Inhibition of HMGR reductase by secondary metabolites. **Table 6S.** Inhibition of XO by secondary metabolites. **Table 7S.** Prediction of toxicity of secondary metabolites inhibiting metabolic enzymes using ProTox-II. **Table 8S (a):** ADMET properties of XO inhibitors by pKCSM server. **Table 8S (b):** ADMET properties of HMGR inhibitors by pKCSM server. **Table 9S**. Crude natural product extracts showing the inhibitory activity against HMGR and XO. **Table 10S.** Gold Fitness score and Protein-Ligand Interactions of Protein ID: 1HWK, HMG-CoA Reductase Inhibition, and Protein ID: 1N5X Xanthine Oxidase Inhibition. The Gold Fitness score, interacting residues, type of interaction, bond length between residues, and ligands are shown. **Table 11S:** Binding Free Energy calculations for XO with inhibitors using semi-empirical method. **Table 12S:** Binding Free Energy calculations for HMGR with inhibitors using semi-empirical method. **Figure** **1****S**. 2D (upper) and 3D (lower) interactions of HMG-CoA Reductase (PDB ID: 1HWK) with atorvastatin (Fitness score of 73.24). **Figure** **2****S.** 2D (upper) and 3D (lower) interactions of HMG-CoA Reductase with simvastatin (Fitness score of 56.81). **Figure** **3****S.** 2D (upper) and 3D (lower) interactions of HMG-CoA Reductase with lovastatin (Fitness score of 41.36). **Fig. 4S.** 2D (upper) and 3D (lower) interactions of HMG-CoA Reductase with pravastatin (Fitness score of 54.83). **Fig. 5S.** 2D (upper) and 3D (lower) interactions of Xanthine Oxidase (PDB ID: 1N5X) with febuxostat (Fitness score of 64.53). **Fig. 6S.** 2D (upper) and 3D (lower) interactions of Xanthine Oxidase with allopurinol (Fitness score of 46.16). **Fig. 7S.** 2D (upper) and 3D (lower) interactions of Xanthine Oxidase with probenecid (Fitness score of 57.75).

## Data Availability

The datasets used and/or analyzed during the current study are available from the corresponding author on reasonable request.

## Abbreviations

ACAT-2: Acyl-CoA cholesterol acyltransferase 2
AI: Artificial intelligence
ANGPTL3: Angiopoietin-like protein 3
ATP: Adenosine triphosphate
BBB: Blood-brain barrier
CVDs: Cardiovascular diseases
CYP: Cytochrome P450
DGAT-2: Diglyceride acyltransferase 2
ER: Endoplasmic reticulum
GOLD: Genetic optimization for ligand docking
HDL: High-density lipoproteins
HMG-CoA: β-Hydroxy β-methylglutaryl-coenzyme A
HMGR: HMG-CoA reductase
IHD: Ischemic heart disease
kDa: Kilodalton
LDL: Low-density lipoprotein
MACE: Major adverse cardiovascular events
MOE: Molecular Operating Environment
NOS: Nitric oxide synthase
PCSK9: Proprotein convertase subtilisin kexin type 9
ROS: Reactive oxygen species
SREBPs: Sterol regulatory element-binding proteins
TC: Total cholesterol
TG: Triglyceride
TPSA: Total polar surface area
VLDL: Very low-density lipoprotein
WHO: World Health Organization
XO: Xanthine oxidase
